# Sherman’s and related inequalities with applications in information theory

**DOI:** 10.1186/s13660-018-1692-0

**Published:** 2018-04-24

**Authors:** S. Ivelić Bradanović, N. Latif, Ð. Pečarić, J. Pečarić

**Affiliations:** 1 Faculty of Civil Engineering, Architecture And Geodesy, University of Split, Split, Croatia; 2 Department of General Studies, Jubail Industrial College, Jubail Industrial City, Kingdom of Saudi Arabia; 3 Catholic University of Croatia, Zagreb, Croatia; 4 Faculty of Textile Technology Zagreb, University of Zagreb, Zagreb, Croatia; 5 RUDN University, Moscow, Russia

**Keywords:** 26D15, 26D20, 26D99, 94A17, Sherman theorem, Majorization inequality, Jensen inequality, Green function, Abel–Gontscharoff interpolating polynomial, *n*-convex function, Entropy, Information theory, *ϕ*-divergence, Zipf–Mandelbrot law

## Abstract

In this paper we give extensions of Sherman’s inequality considering the class of convex functions of higher order. As particular cases, we get an extended weighted majorization inequality as well as Jensen’s inequality which have direct connection to information theory. We use the obtained results to derive new estimates for Shannon’s and Rényi’s entropy, information energy, and some well-known measures between probability distributions. Using the Zipf–Mandelbrot law, we introduce new functionals to derive some related results.

## Introduction and preliminaries

We start with a brief overview of divided differences and *n*-convex functions and give some basic results from the majorization theory.

An *n*th order divided difference of a function $\phi:[\alpha,\beta ]\rightarrow\mathbb{R}$ at distinct points $x_{0},x_{1},\ldots, x_{n}\in {}[\alpha,\beta]$ may be defined recursively by

$$\begin{aligned} &[ x_{i};\phi ] =\phi(x_{i}),\quad i=0,\ldots,n, \\ &[ x_{0},\ldots,x_{n};\phi] =\frac{[x_{1},\ldots,x_{n};\phi ]-[x_{0},\ldots,x_{n-1};\phi]}{x_{n}-x_{0}}. \end{aligned}$$

The value $[x_{0},\ldots,x_{n};\phi]$ is independent of the order of the points $x_{0},\ldots,x_{n}$.

A function *ϕ* is *n*-convex on $[\alpha,\beta]$ if

$${}[ x_{0},x_{1},\ldots,x_{n};\phi]\geq0 $$

holds for all choices of $(n+1)$ distinct points $x_{i}\in{}[\alpha ,\beta]$, $i=0,\ldots,n$.

### Remark 1

From this definition it follows that 1-convex function is an increasing function and 2-convex function is just a convex function. If $\phi^{(n)}$ exists, then *ϕ* is *n*-convex iff $\phi^{(n)}\geq0$. Also, if *ϕ* is *n*-convex for $n\geq2$, then $\phi^{(k)}$ exists and *ϕ* is $(n-k)$-convex for $1\leq k\leq n-2$. For more information, see [1].

For two vectors $\mathbf{x},\mathbf{y}\in{}[\alpha,\beta]^{l}$, let $x_{[i]},y_{[i]}$ denote the *i*th largest entries of **x** and **y**, respectively. It is well known that

$$\sum_{i=1}^{m}y_{[i]}\leq\sum _{i=1}^{m}x_{[i]}\quad\text{for }m=1,2, \ldots ,l-1\quad\text{and}\quad\sum_{i=1}^{l}x_{i}= \sum_{i=1}^{l}y_{i}, $$

i.e., we say that **x** majorizes $\mathbf{y,}$ in symbol $\mathbf{y\prec x}$, iff

$$\mathbf{y}=\mathbf{xA}$$

for some doubly stochastic matrix $\mathbf{A}=(a_{ij})\in\mathcal{M}_{l}(\mathbb{R})$, i.e., a matrix with nonnegative entries and rows and columns sums equal to 1. Moreover, $\mathbf{y\prec x}$ implies

$${ \sum_{i=1}^{l}} \phi(y_{i})\leq { \sum _{i=1}^{l}} \phi(x_{i}) $$

for every continuous convex function $\phi:[\alpha,\beta]\rightarrow \mathbb{R}$. This result, obtained by Hardy et al. (1929 [2]), is well known as a majorization inequality and plays an important role in the study of majorization theory.

Sherman [3] considered the weighted concept of majorization

$$( \mathbf{a}, \mathbf{y} ) \prec ( \mathbf{b}, \mathbf{x} ) $$

between two vectors $\mathbf{x}=(x_{1},\ldots,x_{m})\in{}[\alpha ,\beta]^{m}$ and $\mathbf{y}=(y_{1},\ldots,y_{l})\in{}[\alpha,\beta]^{l}$ with nonnegative weights $\mathbf{a}=(a_{1},\ldots,a_{m})$ and $\mathbf{b}=(b_{1},\ldots ,b_{l})$. The concept of weighted majorization is defined by the assumption of existence of a matrix $\mathbf{A}=(a_{ij})\in\mathcal{M}_{lm}(\mathbb{R})$ such that

1.1 $$\begin{aligned} &a_{ij} \geq0\quad\text{for all }i,j, \end{aligned}$$

1.2 $$\begin{aligned} &{ \sum_{j=1}^{m}} a_{ij} =1,\quad i=1,\ldots,l, \end{aligned}$$

1.3 $$\begin{aligned} &a_{j} ={ \sum _{i=1}^{l}} b_{i}a_{ij},\quad j=1,\ldots,m, \end{aligned}$$

1.4 $$\begin{aligned} &y_{i} ={ \sum _{j=1}^{m}} x_{j}a_{ij},\quad i=1,\ldots,l. \end{aligned}$$

The matrix $\mathbf{A}=(a_{ij})\in\mathcal{M}_{lm}(\mathbb{R})$ with conditions (1.1) and (1.2) is called row stochastic matrix. Sherman proved that under conditions (1.1)–(1.4) for every convex function $\phi:[\alpha,\beta]\rightarrow\mathbb{R}$, the inequality

1.5 $$ \sum_{i=1}^{l}b_{i} \phi(y_{i})\leq\sum_{j=1}^{m}a_{j} \phi(x_{j}) $$

holds. We can write conditions (1.3) and (1.4) in the form

1.6 $$ \mathbf{a}=\mathbf{bA}\quad\text{and}\quad\mathbf{y}=\mathbf{xA}^{T}, $$

where $\mathbf{A}^{T}$ denotes the transpose matrix.

As a special case of Sherman’s inequality, when $l=m$ and $a_{j}=b_{i}$, for all $i,j=1,\ldots,m$, we get the weighted version of majorization inequality

$$\sum_{i=1}^{m}a_{i} \phi(y_{i})\leq\sum_{i=1}^{m}a_{i} \phi(x_{i}). $$

Putting $\sum_{i=1}^{m}a_{i}=1$ and $y_{1}=y_{2}=\cdots=y_{m}=\sum_{i=1}^{m}a_{i}x_{i}$, we get Jensen’s inequality in the form

1.7 $$ \phi \Biggl( { \sum_{i=1}^{m}} a_{i}x_{i} \Biggr) \leq { \sum_{i=1}^{m}} a_{i} \phi(x_{i}). $$

We can get Jensen’s inequality (1.7) directly from (1.5) by setting $l=1$ and $\mathbf{b}=(1)$.

The concept of majorization has a large number of appearances in many different fields of applications, particular in many branches of mathematics. A complete and superb reference on the subject is the monograph [4], and many results from the theory of majorization are directly or indirectly inspired by it. In this paper we give extensions of Sherman’s inequality by considering the class of convex functions of higher order. As a particular case, we get an extension of weighted majorization inequality and Jensen’s inequality which can be used to derive some new estimates for some entropies and measures between probability distributions. Also, we use the Zipf–Mandelbrot law to illustrate the obtained results.

## Some technical lemmas

In this section we present two technical lemmas that give us two identities which will be very useful for us to obtain main results.

Let us consider the function $G:[\alpha,\beta]\times{}[\alpha ,\beta]\rightarrow\mathbb{R}$ defined by

2.1 $$ G(x,y)= \textstyle\begin{cases} \frac{(x-\beta)(y-\alpha)}{\beta-\alpha}, & \alpha\leq y\leq x,\\ \frac{(y-\beta)(x-\alpha)}{\beta-\alpha}, & x\leq y\leq\beta, \end{cases} $$

which presents Green’s function of the boundary value problem

$$z^{\prime\prime}=0, \qquad z(\alpha)=z(\beta)=0. $$

This function is convex and continuous with respect to both variables *x* and *y*.

Integration by parts easily yields that, for any function $\phi\in C^{2}([\alpha,\beta])$, the following holds:

2.2 $$ \phi(t)=\frac{\beta-t}{\beta-\alpha}\phi(\alpha)+\frac{t-\alpha}{\beta -\alpha }\phi(\beta)+ \int_{\alpha}^{\beta}G(t,y)\phi^{\prime\prime}(y)\,dy. $$

Applying (2.2) to Sherman’s difference $\sum_{j=1}^{m}a_{j}\phi(x_{j})-\sum_{i=1}^{l}b_{i}\phi(y_{i})$, we obtain the first identity.

### Lemma 1

*Let* $\mathbf{x}\in{}[\alpha,\beta]^{m}$, $\mathbf{y}\in {}[\alpha,\beta]^{l}$, $\mathbf{a}\in\mathbb{R}^{m}$, *and* $\mathbf{b}\in\mathbb{R}^{l}$ *be such that* (1.6) *holds for some matrix* $\mathbf{A}\in\mathcal{M}_{lm}(\mathbb{R})$ *with* ${ \sum_{j=1}^{m}} a_{ij}=1$, $i=1,\ldots,l$. *Let* *G* *be defined by* (2.1). *Then*, *for every function* $\phi\in C^{2}([\alpha,\beta])$, *the following identity holds*:

2.3 $$ \sum_{j=1}^{m}a_{j} \phi(x_{j})-\sum_{i=1}^{l}b_{i} \phi (y_{i})= \int_{\alpha}^{\beta} \Biggl( \sum _{j=1}^{m}a_{j}G ( x_{j},y ) - \sum_{i=1}^{l}b_{i}G ( y_{i},y ) \Biggr) \phi^{\prime\prime}(y)\,dy. $$

### Proof

Using (2.2) in Sherman’s difference, we have

$$\begin{aligned} & \sum_{j=1}^{m}a_{j} \phi(x_{j})-\sum_{i=1}^{l}b_{i} \phi (y_{i}) \\ & \quad =\frac{\beta\phi(\alpha)-\alpha\phi(\beta)}{\beta-\alpha} \Biggl( \sum_{j=1}^{m}a_{j}- \sum_{i=1}^{l}b_{i} \Biggr) + \frac {\phi(\beta)-\phi(\alpha)}{\beta-\alpha} \Biggl( \sum_{j=1}^{m}a_{j}x_{j}- \sum_{i=1}^{l}b_{i}y_{i} \Biggr) \\ &\qquad{} + \int_{\alpha}^{\beta} \Biggl( \sum _{j=1}^{m}a_{j}G ( x_{j},y ) - \sum_{i=1}^{l}b_{i}G ( y_{i},y ) \Biggr) \phi^{\prime\prime}(y)\,dy. \end{aligned}$$

Since (1.3) and (1.4) hold, then we have

$$\sum_{j=1}^{m}a_{j}-\sum _{i=1}^{l}b_{i}=0\quad\text{and}\quad\sum _{j=1}^{m}a_{j}x_{j}- \sum_{i=1}^{l}b_{i}y_{i}=0, $$

i.e., we get identity (2.3). □

We use the Abel–Gontscharoff interpolation for two points with integral remainder to obtain another identity.

Let $n,k\in\mathbb{N}$, $n\geq2$, $0\leq k\leq n-1$, and $\phi\in C^{n}([\alpha,\beta])$. Then

2.4 $$ \phi(t)=P_{\mathrm{AG}}(t)+e_{\mathrm{AG}} ( t ), $$

where

$$P_{\mathrm{AG}} ( t ) =\sum_{s=0}^{k} \frac{ ( t-\alpha ) ^{s}}{s!}\phi^{(s)}(\alpha)+\sum_{r=0}^{n-k-2} \Biggl[ \sum_{s=0}^{r}\frac { ( t-\alpha ) ^{k+1+s} ( \alpha-\beta ) ^{r-s}}{ ( k+1+s ) ! ( r-s ) !} \Biggr] \phi^{(k+1+r)}(\beta) $$

is the Abel–Gontscharoff interpolating polynomial for two points of degree $n-1$, and the remainder is given by

$$e_{\mathrm{AG}} ( t ) = \int_{\alpha}^{\beta}G_{n}(t,u) \phi^{(n)}(u)\,du, $$

where

2.5 $$ G_{n, k}(t,u)=\frac{1}{(n-1)!}\textstyle\begin{cases}{ \sum_{s=0}^{k}} \binom{n-1}{s} ( t-\alpha ) ^{s} ( \alpha-u ) ^{n-s-1}, & \alpha\leq u\leq t;\\ -{ \sum_{s=k+1}^{n-1}} \binom{n-1}{s} ( t-\alpha ) ^{s} ( \alpha-u ) ^{n-s-1}, & t\leq u\leq\beta. \end{cases} $$

Further, for $\alpha\leq u,t\leq\beta$, the following inequalities hold:

2.6 $$\begin{aligned} \begin{aligned} &(-1)^{n-k-1}\frac{\partial^{s}G_{n, k}(t,u)}{\partial t^{s}} \geq0,\quad 0\leq s\leq k, \\ &(-1)^{n-s}\frac{\partial^{s}G_{n, k}(t,u)}{\partial t^{s}} \geq 0,\quad k+1\leq s\leq n-1. \end{aligned} \end{aligned}$$

For more information, see [5].

Now we use interpolation (2.4) on $\phi^{\prime\prime}$ to obtain the second identity.

### Lemma 2

*Let* $\mathbf{x}\in{}[\alpha,\beta]^{m}$, $\mathbf{y}\in{}[\alpha,\beta]^{l}$, $\mathbf{a}\in\mathbb{R}^{m}$, *and* $\mathbf{b}\in\mathbb{R}^{l}$ *be such that* (1.6) *holds for some matrix* $\mathbf{A}\in\mathcal{M}_{lm}(\mathbb{R})$ *with* ${ \sum_{j=1}^{m}} a_{ij}=1$, $i=1,\ldots,l$. *Let* $n,k\in\mathbb{N}$, $n\geq4$, $0\leq k\leq n-1$, *and* *G*, $G_{n, k}$ *be defined by* (2.1), (2.5), *respectively*. *Then*, *for every function* $\phi\in C^{n}([\alpha,\beta])$, *the following identity holds*:

2.7 $$\begin{aligned} & \sum_{j=1}^{m}a_{j} \phi(x_{j})-\sum_{i=1}^{l}b_{i} \phi (y_{i}) \\ &\quad =\sum_{s=0}^{k}\frac{\phi^{(s+2)}(\alpha)}{s!} \int_{\alpha}^{\beta } \Biggl( \sum _{j=1}^{m}a_{j}G ( x_{j},y ) - \sum_{i=1}^{l}b_{i}G ( y_{i},y ) \Biggr) ( y-\alpha ) ^{s}\,dy \\ &\qquad{} +\sum_{r=0}^{n-k-4}\sum _{s=0}^{r}\frac{(-1)^{r-s}(\beta-\alpha)^{r-s}}{(k+1+s)!(r-s)!}\phi^{(k+3+r)}( \beta) \\ & \qquad{}\times \int_{\alpha}^{\beta} \Biggl( \sum _{j=1}^{m}a_{j}G ( x_{j},y ) - \sum_{i=1}^{l}b_{i}G ( y_{i},y ) \Biggr) ( y-\alpha ) ^{k+1+s}\,dy \\ & \qquad{}+ \int_{\alpha}^{\beta} \int_{\alpha}^{\beta} \Biggl( \sum _{j=1}^{m}a_{j}G ( x_{j},y ) -\sum_{i=1}^{l}b_{i}G ( y_{i},y ) \Biggr) G_{n-2, k}(y,u)\phi^{(n)}(u)\,du \,dy. \end{aligned}$$

### Proof

If we apply formula (2.4) to a function $\phi^{\prime\prime}$, it implies substitution of *n* with $n-2$ in (2.4), and we get

2.8 $$\begin{aligned} & \phi^{\prime\prime}(y) \\ &\quad =\sum_{s=0}^{k}\frac{ ( y-\alpha ) ^{s}}{s!}\phi ^{(s+2)}(\alpha)+\sum_{r=0}^{n-k-4} \Biggl[ \sum_{s=0}^{r}\frac{ ( y-\alpha ) ^{k+1+s} ( \alpha-\beta ) ^{r-s}}{ ( k+1+s ) ! ( r-s ) !} \Biggr] \phi^{(k+3+r)}(\beta) \\ &\qquad{} + \int_{\alpha}^{\beta}G_{n-2, k}(y,u) \phi^{(n)}(u)\,du. \end{aligned}$$

Using (2.8) in (2.3), we obtain the required result. □

## Extensions of Sherman’s inequality

We start this section with an extension of Sherman’s inequality to a more general class of *n*-convex functions.

### Theorem 1

*Let* $\mathbf{x}\in{}[\alpha,\beta]^{m}$, $\mathbf{y}\in{}[\alpha,\beta]^{l}$, $\mathbf{a}\in\mathbb{R}^{m}$, *and* $\mathbf{b}\in\mathbb{R}^{l}$ *be such that* (1.6) *holds for some matrix* $\mathbf{A}\in\mathcal{M}_{lm}(\mathbb{R})$ *with* ${ \sum_{j=1}^{m}} a_{ij}=1$, $i=1,\ldots,l$. *Let* $n,k\in\mathbb{N}$, $n\geq4$, $0\leq k\leq n-1$, *and* *G*, $G_{n, k}$ *be defined by* (2.1), (2.5), *respectively*. *If* $\phi\in C^{n}([\alpha,\beta])$ *is* *n*-*convex and*

3.1 $$ \int_{\alpha}^{\beta} \Biggl( \sum _{j=1}^{m}a_{j}G ( x_{j},y ) -\sum_{i=1}^{l}b_{i}G ( y_{i},y ) \Biggr) G_{n-2, k}(y,u)\,dy\geq0,\quad\textit{for all }u \in{}[\alpha,\beta], $$

*then*

3.2 $$\begin{aligned} & \sum_{j=1}^{m}a_{j} \phi(x_{j})-\sum_{i=1}^{l}b_{i} \phi (y_{i}) \\ & \quad\geq\sum_{s=0}^{k}\frac{\phi^{(s+2)}(\alpha)}{s!} \int_{\alpha}^{\beta } \Biggl( \sum _{j=1}^{m}a_{j}G ( x_{j},y ) - \sum_{i=1}^{l}b_{i}G ( y_{i},y ) \Biggr) ( y-\alpha ) ^{s}\,dy \\ &\qquad{} +\sum_{r=0}^{n-k-4}\sum _{s=0}^{r}\frac{(-1)^{r-s}(\beta-\alpha)^{r-s}}{(k+1+s)!(r-s)!}\phi^{(k+3+r)}( \beta) \\ & \qquad{}\times \int_{\alpha}^{\beta} \Biggl( \sum _{j=1}^{m}a_{j}G ( x_{j},y ) - \sum_{i=1}^{l}b_{i}G ( y_{i},y ) \Biggr) ( y-\alpha ) ^{k+1+s}\,dy. \end{aligned}$$

*If the reverse inequality in* (3.1) *holds*, *then also the reverse inequality in* (3.2) *holds*.

### Proof

Under the assumptions of the theorem, identity (2.7) holds. Since *ϕ* is *n*-convex, then $\phi^{(n)}\geq0$ on $[\alpha,\beta]$. Therefore, if (3.1) is satisfied, then inequality (3.2) holds. □

### Remark 2

Since we have $(-1)^{n-k-3}G_{n-2,k}(y,u)\geq0$ by (2.6), then in case $n-k$ is odd, instead assumption (3.1), it is enough to assume that

$$\sum_{j=1}^{m}a_{j}G ( x_{j},y ) -\sum_{i=1}^{l}b_{i}G ( y_{i},y ) \geq0, \quad\text{for } y\in{}[ \alpha,\beta]. $$

The following extension of Sherman’s inequality, under Sherman’s condition of nonnegativity of vectors $\mathbf{a,b}$, and matrix **A**, also holds.

### Theorem 2

*Let* $\mathbf{x}\in{}[\alpha,\beta]^{m}$, $\mathbf{y}\in{}[\alpha,\beta]^{l}$, $\mathbf{a}\in{}[0,\infty )^{m}$, *and* $\mathbf{b}\in{}[0,\infty)^{l}$ *be such that* (1.6) *holds for some row stochastic matrix* $\mathbf{A}\in\mathcal{M}_{lm}(\mathbb{R})$. *Let* $n,k\in\mathbb{N}$, $n\geq4$, $0\leq k\leq n-1$, *be such that* $n-k$ *is odd*. *Let* *G*, $G_{n,k}$ *be defined by* (2.1), (2.5), *respectively*, *and* $\phi\in C^{n}([\alpha ,\beta])$ *be* *n*-*convex*. *Then inequality* (3.2) *holds*.

### Proof

Since by (2.6) we have $(-1)^{n-k-3}G_{n-2,k}(y,u)\geq0$, then, when $n-k$ is odd, we have

$$G_{n-2,k}(y,u)\geq0. $$

Further, $G(\cdot,y),y\in{}[\alpha,\beta]$, is convex on $[\alpha ,\beta]$, and by Sherman’s inequality, we have

$$\sum_{j=1}^{m}a_{j}G ( x_{j},y ) -\sum_{i=1}^{l}b_{i}G ( y_{i},y ) \geq0. $$

Combining these two facts, assumption (3.1) is satisfied. Hence by Theorem 1, inequality (3.2) holds. □

### Remark 3

In case $n-k$ is even, then the reverse inequality in (3.1) holds, i.e., the reverse inequality in (3.2) holds.

### Theorem 3

*Let all the assumptions of Theorem *2 *be satisfied*.

(i) *If* $\phi^{(s+2)}(\alpha)\geq0$ *for each* $s=0,\ldots,k$ *and* $(-1)^{r-s}\phi ^{(k+3+r)}(\beta)\geq0$ *for each* $r=0,\ldots,n-k-4$ *and* $s=0,\ldots,r$, *then*
  3.3 $$\begin{aligned} & \sum_{j=1}^{m}a_{j} \phi(x_{j})-\sum_{i=1}^{l}b_{i} \phi (y_{i}) \\ & \quad\geq\sum_{s=0}^{k}\frac{\phi^{(s+2)}(\alpha)}{s!} \int_{\alpha}^{\beta } \Biggl( \sum _{j=1}^{m}a_{j}G ( x_{j},y ) - \sum_{i=1}^{l}b_{i}G ( y_{i},y ) \Biggr) ( y-\alpha ) ^{s}\,dy \\ &\qquad{} +\sum_{r=0}^{n-k-4}\sum _{s=0}^{r}\frac{(-1)^{r-s}(\beta-\alpha)^{r-s}}{(k+1+s)!(r-s)!}\phi^{(k+3+r)}( \beta) \\ &\qquad{}\times \int_{\alpha}^{\beta} \Biggl( \sum _{j=1}^{m}a_{j}G ( x_{j},y ) - \sum_{i=1}^{l}b_{i}G ( y_{i},y ) \Biggr) ( y-\alpha ) ^{k+1+s}\,dy \\ & \quad\geq0. \end{aligned}$$
(ii) *If the function*
  3.4 $$\begin{aligned} F(\cdot) ={}&\sum_{s=0}^{k} \frac{\phi^{(s+2)}(\alpha)}{s!} \int_{\alpha }^{\beta}G(\cdot,y) ( y-\alpha ) ^{s} \,dy \\ &{} +\sum_{r=0}^{n-k-4}\sum _{s=0}^{r}\frac{ ( -1 ) ^{r-s} ( \beta-\alpha ) ^{r-s}}{ ( k+1+s ) ! ( r-s ) !}\phi^{(k+3+r)}(\beta) \int_{\alpha}^{\beta}G(\cdot,y) ( y-\alpha ) ^{k+1+s} \,dy \end{aligned}$$
  *is convex on* $[\alpha,\beta]$, *then* (3.3) *holds*.

### Proof

(i) Under the assumptions,the nonnegativity of the right-hand side of (3.2) is obvious, i.e., the double inequality (3.3) holds.

(ii) The right-hand side of (3.2) can be written in the form $\sum_{j=1}^{m}a_{j}F ( x_{j} ) -\sum_{i=1}^{l}b_{i}F ( y_{i} ) $. So, if *F* is convex, then by Sherman’s inequality we have

$$\sum_{j=1}^{m}a_{j}F ( x_{j} ) -\sum_{i=1}^{l}b_{i}F ( y_{i} ) \geq0, $$

i.e., we again get the nonnegativity of the right-hand side of (3.2), which we need to prove. □

### Remark 4

Note that inequality (3.3) includes a new lower bound for Sherman’s difference in the form

3.5 $$\begin{aligned} A_{n}(\phi;\alpha,\beta) ={}&\sum_{s=0}^{k} \frac{\phi^{(s+2)}(\alpha)}{s!} \int_{\alpha}^{\beta} \Biggl( \sum _{j=1}^{m}a_{j}G ( x_{j},y ) - \sum_{i=1}^{l}b_{i}G ( y_{i},y ) \Biggr) ( y-\alpha ) ^{s}\,dy \\ &{} +\sum_{r=0}^{n-k-4}\sum _{s=0}^{r}\frac{(-1)^{r-s}(\beta-\alpha)^{r-s}}{(k+1+s)!(r-s)!}\phi^{(k+3+r)}( \beta) \\ &{}\times \int_{\alpha}^{\beta} \Biggl( \sum _{j=1}^{m}a_{j}G ( x_{j},y ) - \sum_{i=1}^{l}b_{i}G ( y_{i},y ) \Biggr) ( y-\alpha ) ^{k+1+s}\,dy. \end{aligned}$$

Specially, for $n=4$, $k=1$, the lower bound has the form

3.6 $$ \int_{\alpha}^{\beta} \Biggl( { \sum _{j=1}^{m}} a_{j}G ( x_{j},y ) -{ \sum _{i=1}^{l}} b_{i}G ( y_{i},y ) \Biggr) \bigl(\phi^{\prime\prime}(\alpha )+ \phi^{\prime\prime\prime}(\alpha) ( y-\alpha ) \bigr)\,dy. $$

Using notation $\Vert \cdot \Vert _{p}$ for the standard *p*-norm and applying the well-known Hölder inequality, we obtain the following result.

### Theorem 4

*Let* $p,q$ *be a pair of conjugate exponents*, *i*.*e*., $1< p,q<\infty $, $1/p+1/q=1$. *Let* $\mathbf{x}\in{}[\alpha,\beta]^{m}$, $\mathbf{y}\in{}[\alpha,\beta]^{l}$, $\mathbf{a}\in\mathbb{R}^{m}$, *and* $\mathbf{b}\in\mathbb{R}^{l}$ *be such that* (1.6) *holds for some matrix* $\mathbf{A}\in\mathcal{M}_{lm}(\mathbb{R})$ *with* ${ \sum_{j=1}^{m}} a_{ij}=1$, $i=1,\ldots,l$. *Let* $n,k\in\mathbb{N}$, $n\geq4$, $0\leq k\leq n-1$, $\phi\in C^{n}([\alpha,\beta])$, *and* *G*, $G_{n, k}$ *be defined by* (2.1), (2.5), *respectively*. *Then*

$$\begin{aligned} & \Biggl\vert \sum_{j=1}^{m}a_{j} \phi ( x_{j} ) -\sum_{i=1}^{l}b_{i} \phi ( y_{i} ) -A_{n}(\phi;\alpha,\beta) \Biggr\vert \\ & \quad\leq \bigl\Vert \phi^{(n)} \bigr\Vert _{p} \Biggl( \int_{\alpha}^{\beta } \Biggl( \int_{\alpha}^{\beta} \Biggl( { \sum _{j=1}^{m}} a_{j}G ( x_{j},y ) -{ \sum _{i=1}^{l}} b_{i}G ( y_{i},y ) \Biggr) G_{n-2, k} ( y,u ) \,dy \Biggr) ^{q}\,du \Biggr) ^{\frac{1}{q}}, \end{aligned}$$

*where* $A_{n}(\phi;\alpha,\beta)$ *is defined by* (3.5).

### Proof

Under the assumptions of the theorem, identity (2.7) holds. Applying Hölder’s inequality to (2.7), we get

$$\begin{aligned} & \Biggl\vert \sum_{j=1}^{m}a_{j} \phi ( x_{j} ) -\sum_{i=1}^{l}b_{i} \phi ( y_{i} ) -A_{n}(\phi;\alpha,\beta) \Biggr\vert \\ & \quad= \Biggl\vert \int_{\alpha}^{\beta} \int_{\alpha}^{\beta} \Biggl( \sum _{j=1}^{m}a_{j}G ( x_{j},y ) - \sum_{i=1}^{l}b_{i}G ( y_{i},y ) \Biggr) G_{n-2, k}(y,u)\phi^{(n)}(u) \,du\,dy \Biggr\vert \\ &\quad \leq \bigl\Vert \phi^{(n)} \bigr\Vert _{p} \Biggl( \int_{\alpha}^{\beta } \Biggl( \int_{\alpha}^{\beta} \Biggl( { \sum _{j=1}^{m}} a_{j}G ( x_{j},y ) -{ \sum _{i=1}^{l}} b_{i}G ( y_{i},y ) \Biggr) G_{n-2, k} ( y,u ) \,dy \Biggr) ^{q}\,du \Biggr) ^{\frac{1}{q}}. \end{aligned}$$

 □

As a direct consequence of the previous results, choosing $n=4$ and $k=1$, we get the following corollary.

### Corollary 1

*Let* $p,q$ *be a pair of conjugate exponents*, *i*.*e*., $1< p,q<\infty$, $1/p+1/q=1$. *Let* *G* *be defined by* (2.1), $\mathbf{x}\in{}[\alpha,\beta]^{m}$, $\mathbf{y}\in{}[ \alpha,\beta]^{l}$, $\mathbf{a}\in{}[0,\infty)^{m}$, *and* $\mathbf{b}\in{}[0,\infty)^{l}$ *be such that* (1.6) *holds for some row stochastic matrix* $\mathbf{A}\in\mathcal{M}_{lm}(\mathbb{R})$. *If* $\phi\in C^{4}([\alpha,\beta])$ *is* 4-*convex*, *then*

3.7 $$\begin{aligned} 0 & \leq\sum_{j=1}^{m}a_{j} \phi(x_{j})-\sum_{i=1}^{l}b_{i}\phi(y_{i})- \int_{\alpha}^{\beta}\mathcal{G}(y) \bigl( \phi^{\prime\prime}(\alpha)+\phi^{\prime\prime\prime}(\alpha) ( y-\alpha ) \bigr)\,dy \\ & \leq \bigl\Vert \phi^{(4)} \bigr\Vert _{p} \biggl( \int_{\alpha}^{\beta } \biggl( \int_{\alpha}^{\beta}\mathcal{G}(y)G_{2, 1} ( y,u ) \,dy \biggr) ^{q}\,du \biggr) ^{\frac{1}{q}}, \end{aligned}$$

*where* $\mathcal{G}(y)={ \sum_{j=1}^{m}} a_{j}G ( x_{j},y ) -{ \sum_{i=1}^{l}} b_{i}G ( y_{i},y ) $ *and*

3.8 $$ G_{2, 1}(y,u)=\textstyle\begin{cases} y-u, & \alpha\leq y\leq u,\\ 0, & u\leq y\leq\beta. \end{cases} $$

### Remark 5

Specially, if we set $l=m$ and $a_{j}=b_{i}$ for each $i,j=1,\ldots,l$, from the previous result, as a direct consequence, we obtain the following extension of majorization inequality:

3.9 $$\begin{aligned} 0 & \leq\sum_{i=1}^{m}a_{i} \phi(x_{i})-\sum_{i=1}^{m}a_{i}\phi(y_{i})- \int_{\alpha}^{\beta}\mathcal{G}(y) \bigl( \phi^{\prime\prime}(\alpha)+\phi^{\prime\prime\prime}(\alpha) ( y-\alpha ) \bigr)\,dy \\ & \leq \bigl\Vert \phi^{(4)} \bigr\Vert _{p} \biggl( \int_{\alpha}^{\beta } \biggl( \int_{\alpha}^{\beta}\mathcal{G}(y)G_{2, 1} ( y,u ) \,dy \biggr) ^{q}\,du \biggr) ^{\frac{1}{q}}, \end{aligned}$$

where $\mathcal{G}(y)={ \sum_{i=1}^{m}} a_{i}G ( x_{i},y ) -{ \sum_{i=1}^{m}} a_{i}G ( y_{i},y ) $.

### Remark 6

By setting $l=1$, $\mathbf{b}=(1)$, from (3.7), as a direct consequence, we get the extension of Jensen’s inequality

3.10 $$\begin{aligned} 0 & \leq\sum_{i=1}^{m}a_{i} \phi(x_{i})-\phi \Biggl( { \sum _{i=1}^{m}} a_{i}x_{i} \Biggr) - \int_{\alpha}^{\beta}\mathcal{G}(y) \bigl( \phi^{\prime \prime }(\alpha)+\phi^{\prime\prime\prime}(\alpha) ( y-\alpha ) \bigr) \,dy \\ & \leq \bigl\Vert \phi^{(4)} \bigr\Vert _{p} \biggl( \int_{\alpha}^{\beta } \biggl( \int_{\alpha}^{\beta}\mathcal{G}(y)G_{2, 1} ( y,u ) \,dy \biggr) ^{q}\,du \biggr) ^{\frac{1}{q}}, \end{aligned}$$

where $\mathcal{G}(y)={ \sum_{i=1}^{m}} a_{i}G ( x_{i},y ) -G ( \sum_{i=1}^{m}a_{i}x_{i},y ) $.

## Applications in information theory

Throughout the rest of paper, let $\alpha,\beta$ be positive real numbers such that $0<\alpha<\beta$.

By *X* we denote a discrete random variable with distribution

$$ \begin{pmatrix} x_{1} & x_{2} & \ldots& x_{m}\\ p_{1} & p_{2} & \ldots& p_{m}\end{pmatrix} , $$

where $\mathbf{p}=(p_{1},\ldots,p_{m})$ is a positive probability distribution, i.e., $p_{i}>0$, $i=1,\ldots,m$, with ${ \sum_{i=1}^{m}} p_{i}=1$.

*Shannon entropy* [6] is defined by

$$H(X)=\sum_{i=1}^{m}p_{i}\ln \frac{1}{p_{i}}. $$

It is well known that the maximum possible value of $H(X)$ concerns in terms of the size of $R(X)$, i.e., the inequality

$$0\leq H(X)\leq\ln m $$

holds. Furthermore, $H(X)=0$ iff $p_{i}=1$ for some *i*, and $H(X)=\ln m$ iff $p_{i}=\frac{1}{m}$ for all $i=1,\ldots,m$. Some related results can be found in [7–13].

### Corollary 2

*Let* $p,q$ *be a pair of conjugate exponents*, *i*.*e*., $1< p,q<\infty$, $1/p+1/q=1$. *Let* *G*, $G_{2, 1}$ *be defined by* (2.1), (3.8), *respectively*. *Let* $\boldsymbol{\xi} = ( \xi_{1}, \ldots, \xi_{m} ) \in{}[ \alpha,\beta]^{m}$ *and* $\mathbf{p}= ( p_{1}, \ldots, p_{m} ) $ *be a positive probability distribution*. *Then*

4.1 $$\begin{aligned} 0 & \leq\ln \Biggl( \sum_{i=1}^{m}p_{i} \xi_{i} \Biggr) -\sum_{i=1}^{m}p_{i} \ln\xi_{i}- \int_{\alpha}^{\beta}\mathcal{G}(y) \frac{3\alpha-2y}{\alpha^{3}}\,dy \\ & \leq6 \biggl( \frac{\beta^{1-4p}-\alpha^{1-4p}}{1-4p} \biggr) ^{\frac {1}{p}} \biggl( \int_{\alpha}^{\beta} \biggl( \int_{\alpha}^{\beta}\mathcal{G}(y)G_{2, 1} ( y,u ) \,dy \biggr) ^{q}\,du \biggr) ^{\frac{1}{q}}, \end{aligned}$$

*where* $\mathcal{G}(y)={ \sum_{i=1}^{m}} p_{i}G ( \xi_{i},y ) -G ( { \sum_{i=1}^{m}} p_{i}\xi_{i},y ) $.

### Proof

Substituting $\xi_{i}$ in place of $x_{i}$, $p_{i}$ in place of $a_{i}$ in (3.10) and choosing $\phi(x)=-\ln x$, we obtain (4.1). □

### Corollary 3

*Let* $p,q$ *be a pair of conjugate exponents*, *i*.*e*., $1< p,q<\infty$, $1/p+1/q=1$. *Let* *G*, $G_{2, 1}$ *be defined by* (2.1), (3.8), *respectively*. *Let* **p** *be a positive probability distribution with* $p_{i}^{-1} \in{}[\alpha,\beta]$, $i=1,\ldots,m$. *Then*

$$\begin{aligned} 0 & \leq\ln m-H(X)- \int_{\alpha}^{\beta}\mathcal{G}(y)\frac{3\alpha -2y}{\alpha^{3}}\,dy \\ & \leq6 \biggl( \frac{\beta^{1-4p}-\alpha^{1-4p}}{1-4p} \biggr) ^{\frac{1}{p}} \biggl( \int_{\alpha}^{\beta} \biggl( \int_{\alpha}^{\beta}\mathcal{G}(y)G_{2, 1} ( y,u ) \,dy \biggr) ^{q}\,du \biggr) ^{\frac{1}{q}}, \end{aligned}$$

*where* $\mathcal{G}(y)={ \sum_{i=1}^{m}} p_{i}G ( p_{i}^{-1},y ) -{ \sum_{i=1}^{m}} G ( m,y ) $.

### Proof

If we substitute $\frac{1}{p_{i}}$ in place of $\xi_{i}$ in (4.1), we get the required result. □

*Rényi’s entropy* [14] of order *λ*, $\lambda\in(0,1)\cup(1,\infty)$, is defined by

$$H_{\lambda}(X)=\frac{1}{1-\lambda}\ln \Biggl( \sum _{i=1}^{m}p_{i}^{\lambda} \Biggr). $$

Applying discrete Jensen’s inequality to the convex function $\phi (x)=-\ln x$, we have

$$\ln \Biggl( \sum_{i=1}^{m}p_{i}x_{i} \Biggr) \geq\sum_{i=1}^{m}p_{i} \ln x_{i}. $$

Substituting $p_{i}^{\lambda-1}$ in place of $x_{i}$, we get

$$\ln \Biggl( \sum_{i=1}^{m}p_{i}^{\lambda} \Biggr) \geq(\lambda -1)\sum_{i=1}^{m}p_{i} \ln p_{i},$$

which is equivalent to

$$(1-\lambda)\bigl[H_{\lambda}(X)-H(X)\bigr]\geq0. $$

Specially, we have

$$\begin{aligned} &H_{\lambda}(X) \geq H(X), \quad\lambda\in(0,1), \\ &H_{\lambda}(X) \leq H(X), \quad\lambda\in(1,\infty) \end{aligned}$$

with the equality in case of the uniform distribution, i.e., when $p_{i} =\frac{1}{m},i=1,\ldots,m$.

### Corollary 4

*Let* $p,q$ *be a pair of conjugate exponents*, *i*.*e*., $1< p,q<\infty$, $1/p+1/q=1$. *Let* *G*, $G_{2, 1}$ *be defined by* (2.1), (3.8), *respectively*. *Let* $\lambda\in(0,1)\cup(1,\infty)$ *and* **p** *be a positive probability distribution with* $p_{i}^{\lambda-1}\in{}[\alpha,\beta]$, $i=1,\ldots,m$. *Then*

$$\begin{aligned} 0 & \leq(1-\lambda)\bigl[H_{\lambda}(X)-H(X)\bigr]- \int_{\alpha}^{\beta}\mathcal{G}(y) \frac{3\alpha-2y}{\alpha^{3}}\,dy \\ & \leq6 \biggl( \frac{\beta^{1-4p}-\alpha^{1-4p}}{1-4p} \biggr) ^{\frac{1}{p}} \biggl( \int_{\alpha}^{\beta} \biggl( \int_{\alpha}^{\beta}\mathcal{G}(y)G_{2, 1} ( y,u ) \,dy \biggr) ^{q}\,du \biggr) ^{\frac{1}{q}}, \end{aligned}$$

*where* $\mathcal{G}(y)={ \sum_{i=1}^{m}} p_{i}G ( p_{i}^{\lambda-1},y ) -G ( { \sum_{i=1}^{m}} p_{i}^{\lambda},y ) $.

### Proof

Substituting $p_{i}^{\lambda-1}$ in place of $\xi_{i}$ in (4.1), we obtain the required result. □

The information energy of the random variable *X* is defined by

$$E(X)={ \sum_{i=1}^{m}} p_{i}^{2}. $$

### Corollary 5

*Let* $p,q$ *be a pair of conjugate exponents*, *i*.*e*., $1< p,q<\infty$, $1/p+1/q=1$. *Let* *G*, $G_{2, 1}$ *be defined by* (2.1), (3.8), *respectively*. *Let* $\boldsymbol{\xi} = ( \xi_{1}, \ldots, \xi_{m} ) \in{}[ \alpha,\beta]^{m}$ *and* $\mathbf{p}=p_{1}, \ldots, p_{m}$ *be a positive probability distribution*.

(i) *For* $\lambda\in(0,1)$, *we have*
  4.2 $$\begin{aligned} 0 \leq{}& \Biggl( \sum_{i=1}^{m}p_{i} \xi_{i} \Biggr) ^{\lambda}-\sum _{i=1}^{m}p_{i}\xi_{i}^{\lambda} \\ &{} - \int_{\alpha}^{\beta}\mathcal{G}(y) \bigl( \lambda(1-\lambda )\alpha^{\lambda-2}-\lambda(1-\lambda) (2-\lambda)\alpha^{\lambda-3} ( y- \alpha ) \bigr) \,dy \\ \leq{}&\lambda(1-\lambda) (2-\lambda) (3-\lambda) \biggl( \frac{\beta ^{(\lambda-4)p+1}-\alpha^{(\lambda-4)p+1}}{(\lambda-4)p+1} \biggr) ^{\frac {1}{p}} \\ &{}\times\biggl( \int_{\alpha}^{\beta} \biggl( \int_{\alpha}^{\beta}\mathcal{G}(y)G_{2, 1} ( y,u ) \,dy \biggr) ^{q}\,du \biggr) ^{\frac {1}{q}}, \end{aligned}$$
  *where*
  4.3 $$ \mathcal{G}(y)={ \sum _{i=1}^{m}} p_{i}G ( \xi_{i},y ) -G \Biggl( { \sum _{i=1}^{m}} p_{i} \xi_{i},y \Biggr). $$
(ii) *For* $\lambda\in(1,\infty)$, *we have*
  $$\begin{aligned} 0 \leq{}&\sum_{i=1}^{m}p_{i} \xi_{i}^{\lambda}- \Biggl( \sum_{i=1}^{m}p_{i} \xi_{i} \Biggr) ^{\lambda} \\ & {}- \int_{\alpha}^{\beta}\mathcal{G}(y) \bigl( \lambda(\lambda-1) \alpha ^{\lambda-2}+\lambda(\lambda-1) (\lambda-2)\alpha^{\lambda-3} ( y- \alpha ) \bigr) \,dy \\ \leq{}&\lambda(\lambda-1) \bigl\vert (\lambda-2) (\lambda-3) \bigr\vert \biggl( \frac{\beta^{(\lambda-4)p+1}-\alpha^{(\lambda-4)p+1}}{(\lambda -4)p+1} \biggr) ^{\frac{1}{p}} \\ &{}\times \biggl( \int_{\alpha}^{\beta} \biggl( \int_{\alpha}^{\beta }\mathcal{G}(y)G_{2, 1} ( y,u ) \,dy \biggr) ^{q}\,du \biggr) ^{\frac{1}{q}}, \end{aligned}$$
  *where* $\mathcal{G}(y)$ *is defined by* (4.3).

### Proof

(i) Substituting $\xi_{i}$ in place of $x_{i}$, $p_{i}$ in place of $a_{i}$ in (3.10), and choosing $\phi(x)=-x^{\lambda},\lambda\in(0,1)$, we obtain (4.2).

(ii) Substituting $\xi_{i}$ in place of $x_{i}$, $p_{i}$ in place of $a_{i}$ in (3.10), and choosing $\phi (x)=x^{\lambda }$, $\lambda\in(1,\infty)$, we obtain the required result. □

### Corollary 6

*Let* $p,q$ *be a pair of conjugate exponents*, *i*.*e*., $1< p,q<\infty$, $1/p+1/q=1$. *Let* *G*, $G_{2, 1}$ *be defined by* (2.1), (3.8), *respectively*. *Let* $\mathbf{p}\in{}[\alpha,\beta]^{m}$ *be a positive probability distribution*.

(i) *For* $\lambda\in(0,1)$, *we have*
  4.4 $$\begin{aligned} 0 \leq{}& E^{\lambda}(X)-\exp\bigl[-\lambda H_{\lambda+1}(X) \bigr] \\ &{} - \int_{\alpha}^{\beta}\mathcal{G}(y) \bigl( \lambda(1-\lambda )\alpha^{\lambda-2}-\lambda(1-\lambda) (2-\lambda)\alpha^{\lambda-3} ( y- \alpha ) \bigr) \,dy \\ \leq{}&\lambda(1-\lambda) (2-\lambda) (3-\lambda) \biggl( \frac{\beta ^{(\lambda-4)p+1}-\alpha^{(\lambda-4)p+1}}{(\lambda-4)p+1} \biggr) ^{\frac {1}{p}} \\ &{}\times\biggl( \int_{\alpha}^{\beta} \biggl( \int_{\alpha}^{\beta}\mathcal{G}(y)G_{2, 1} ( y, u ) \,dy \biggr) ^{q}\,du \biggr) ^{\frac{1}{q}}, \end{aligned}$$
  *where*
  4.5 $$ \mathcal{G}(y)={ \sum _{i=1}^{m}} p_{i}G ( p_{i},y ) -G \bigl( E(X),y \bigr). $$
(ii) *For* $\lambda\in(1,\infty)$, *we have*
  $$\begin{aligned} 0 \leq{}& \exp\bigl[-\lambda H_{\lambda+1}(X)\bigr]- \bigl( E(X) \bigr) ^{\lambda} \\ & {}- \int_{\alpha}^{\beta}\mathcal{G}(y) \bigl( \lambda(\lambda-1) \alpha ^{\lambda-2}+\lambda(\lambda-1) (\lambda-2)\alpha^{\lambda-3} ( y- \alpha ) \bigr) \,dy \\ \leq{}&\lambda(\lambda-1) \bigl\vert (\lambda-2) (\lambda-3) \bigr\vert \biggl( \frac{\beta^{(\lambda-4)p+1}-\alpha^{(\lambda-4)p+1}}{(\lambda -4)p+1} \biggr) ^{\frac{1}{p}} \\ &{}\times\biggl( \int_{\alpha}^{\beta} \biggl( \int_{\alpha}^{\beta }\mathcal{G}(y)G_{2, 1} ( y,u ) \,dy \biggr) ^{q}\,du \biggr) ^{\frac{1}{q}}, \end{aligned}$$
  *where* $\mathcal{G}(y)$ *is defined by* (4.5).

### Proof

(i) Substituting $p_{i}$ in place of $\xi_{i}$ in (4.2) and taking into account that

$$\sum_{i=1}^{m}p_{i}^{\lambda}= \exp\bigl[(1-\lambda)H_{\lambda}(X)\bigr], $$

i.e.,

$$\sum_{i=1}^{m}p_{i}^{\lambda+1}= \exp\bigl[-\lambda H_{\lambda+1}(X)\bigr], $$

we get (4.4).

(ii) Similar to (i). □

Let $\mathbf{u},\mathbf{v}$ be two positive probability distributions. The following measures are well known in information theory:

- *Hellinger discrimination*:
  $$h^{2} ( \mathbf{u},\mathbf{v} ) =\frac{1}{2}\sum _{i=1}^{m} ( \sqrt{u_{i}}- \sqrt{v_{i}} ) ^{2}. $$
- $\boldsymbol{\chi^{2}}$-*divergence*:
  $$D_{\chi^{2}} ( \mathbf{u},\mathbf{v} ) =\sum_{i=1}^{m} \frac { ( u_{i}-v_{i} ) ^{2}}{v_{i}}. $$
- *Triangular discrimination*:
  $$\Delta ( \mathbf{u},\mathbf{v} ) =\sum_{i=1}^{m} \frac{ ( u_{i}-v_{i} ) ^{2}}{u_{i}+v_{i}}. $$

In the following results, we consider positive probability distributions $\mathbf{u},\mathbf{v,w}$ with the assumption of existence of a row stochastic matrix $\mathbf{A}\in\mathcal{M}_{m}(\mathbb{R})$ such that

4.6 $$ \mathbf{w}=\mathbf{wA}\quad\text{and}\quad\frac{\mathbf{v}}{\mathbf{w}}= \frac{\mathbf{u}}{\mathbf{w}}\mathbf{A}^{T}, $$

where $\frac{\mathbf{u}}{\mathbf{w}}= ( \frac{u_{1}}{w_{1}},\ldots,\frac{u_{m}}{w_{m}} ) $ and $\frac{\mathbf{v}}{\mathbf {w}}= ( \frac{v_{1}}{w_{1}},\ldots,\frac{v_{m}}{w_{m}} ) $.

### Corollary 7

*Let* $p,q$ *be a pair of conjugate exponents*, *i*.*e*., $1< p,q<\infty$, $1/p+1/q=1$. *Let* *G*, $G_{2, 1}$ *be defined by* (2.1), (3.8), *respectively*. *Let* $\mathbf{u},\mathbf{v,w}$ *be positive probability distributions such that* $\frac{\mathbf{u}}{\mathbf{w}},\frac{\mathbf{v}}{\mathbf{w}}\in{}[ \alpha,\beta]^{m}$ *and* (4.6) *is satisfied for some row stochastic matrix* $\mathbf{A}\in\mathcal{M}_{m}(\mathbb{R})$. *Then*:

(i) 4.7 $$\begin{aligned} 0 & \leq h^{2} ( \mathbf{u},\mathbf{w} ) -h^{2} ( \mathbf{v},\mathbf{w} ) - \int_{\alpha}^{\beta}\mathcal{G}(y) \biggl[ \frac{1}{4}\alpha^{-\frac{3}{2}}-\frac{3}{8}\alpha^{-\frac{5}{2}} ( y-\alpha ) \biggr] \,dy \\ & \leq\frac{15}{16} \biggl[ \frac{2}{2-7p} \bigl( \beta^{\frac{2-7p}{2}}- \alpha^{\frac{2-7p}{2}} \bigr) \biggr] ^{\frac{1}{p}} \biggl( \int _{\alpha }^{\beta} \biggl( \int_{\alpha}^{\beta}\mathcal{G}(y)G_{2, 1} ( y,u ) \,dy \biggr) ^{q}\,du \biggr) ^{\frac{1}{q}}, \end{aligned}$$
  *where*
  4.8 $$ \mathcal{G}(y)={ \sum _{i=1}^{m}} w_{i}G \biggl( \frac{u_{i}}{w_{i}},y \biggr) -{ \sum _{i=1}^{m}} w_{i}G \biggl( \frac{v_{i}}{w_{i}},y \biggr). $$
(ii) 4.9 $$ D_{\chi^{2}} ( \mathbf{u},\mathbf{w} ) -D_{\chi^{2}} ( \mathbf{v}, \mathbf{w} ) =2 \int_{\alpha}^{\beta}\mathcal{G}(u)\,du, $$
  *where* $\mathcal{G}(y)$ *is defined by* (4.8).
(iii) 4.10 $$\begin{aligned} 0 & \leq\Delta ( \mathbf{u},\mathbf{w} ) -\Delta ( \mathbf{v},\mathbf{w} ) - \int_{\alpha}^{\beta}\mathcal{G}(y) \bigl[ 8 ( \alpha+1 ) ^{-3}-24 ( \alpha+1 ) ^{-4}(y-\alpha) \bigr] \,dy \\ & \leq96 \biggl[ \frac{ ( \beta+1 ) ^{1-5p}- ( \alpha +1 ) ^{1-5p}}{1-5p} \biggr] ^{\frac{1}{p}} \biggl( \int_{\alpha }^{\beta } \biggl( \int_{\alpha}^{\beta}\mathcal{G}(y)G_{2, 1} ( y,u ) \,dy \biggr) ^{q}\,du \biggr) ^{\frac{1}{q}}, \end{aligned}$$
  *where* $\mathcal{G}(y)$ *is defined by* (4.8).

### Proof

If we substitute $x_{i}$ by $\frac{u_{i}}{w_{i}}$, $y_{i}$ by $\frac {v_{i}}{w_{i}}$, $a_{i}$ by $w_{i}$ in (3.9) and

(i) take $\phi(x)=\frac{1}{2} ( \sqrt{x}-1 ) ^{2}$, we obtain (4.7).
(ii) take $\phi(x)= ( x-1 ) ^{2}$, we obtain (4.9).
(iii) take $\phi(x)=\frac{ ( x-1 ) ^{2}}{x+1}$, we obtain (4.10).

 □

## Applications as the Zipf–Mandelbrot law

The Zipf–Mandelbrot law is a discrete probability distribution depending on three parameters $m\in\mathbb{N}$, $t\geq0$, and $s>0$ with probability mass function defined by

$$f ( k,m,t,s ) =\frac{1}{(k+t)^{s}H_{m,t,s}}, \quad k=1,2,\ldots,m, $$

where

$$H_{m,t,s}=\sum_{i=1}^{m} \frac{1}{(i+t)^{s}}. $$

When $t=0$, we get so-called Zipf’s law.

The Zipf–Mandelbrot, as well as Zipf’s, law has wide applications in many branches of science as well as linguistics [15], information sciences [16, 17], ecological field studies [18], etc. For more information, see also [15, 19].

We introduce the following definitions of Csiszár divergence for the Zipf–Mandelbrot law. For more information about Csiszár divergence, see [20, 21].

### Definition 1

(Csiszár divergence for Z–M law)

Let $m\in\mathbb{N}$ and $\phi:[\alpha,\beta]\rightarrow\mathbb{R}$ be a function.

- For $t\geq0$ and $s,r_{{1}},\ldots,r_{m}>0$, such that
  $$\frac{1}{r_{i}(i+t)^{s}H_{m,t,s}}\in{}[\alpha,\beta],\quad i=1,\ldots,m, $$
  we define
  $$\hat{I}_{\phi} ( m,t,s,\mathbf{r} ) =\sum_{i=1}^{m}r_{i} \phi \biggl( \frac{1}{r_{{i}}(i+t)^{s}H_{m,t,s}} \biggr). $$
  Specially, when $r_{i}=1$, $i=1,\ldots,m$, we have
  $$\hat{I}_{\phi} ( m,t,s,\mathbf{1} ) =\sum_{i=1}^{m} \phi \biggl( \frac{1}{(i+t)^{s}H_{m,t,s}} \biggr). $$
- For $t, \tilde{t} \geq0$ and $s,\tilde{s}>0$, such that
  $$\frac{(i+\tilde{t})^{\tilde{s}}H_{m,\tilde{t},\tilde {s}}}{(i+t)^{s}H_{m,t,s}}\in{}[\alpha,\beta],\quad i=1,\ldots,m, $$
  we define
  $$\widetilde{I}_{\phi} ( m,t,\tilde{t},s,\tilde{s} ) =\sum _{i=1}^{m}\frac{1}{(i+\tilde{t})^{\tilde{s}}H_{m,\tilde{t},\tilde{s}}}\phi \biggl( \frac{(i+\tilde{t})^{\tilde{s}}H_{m,\tilde{t},\tilde {s}}}{(i+t)^{s}H_{m,t,s}} \biggr). $$

### Corollary 8

*Let* $p,q$ *be a pair of conjugate exponents*, *i*.*e*., $1< p,q<\infty$, $1/p+1/q=1$. *Let* *G*, $G_{2, 1}$ *be defined by* (2.1), (3.8), *respectively*. *Let* $m\in\mathbb{N}$, $t_{1},t_{2},t_{3}\geq0$ *and* $s_{1},s_{2},s_{3}>0$ *be such that*

5.1 $$ \frac {(i+t_{2})^{s_{2}}H_{m,t_{2},s_{2}}}{(i+t_{1})^{s_{1}}H_{m,t_{1},s_{1}}}, \frac{(i+t_{3})^{s_{3}}H_{m,t_{3},s_{3}}}{(i+t_{2})^{s_{2}}H_{m,t_{2},s_{2}}}\in{}[\alpha,\beta],\quad i=1,\ldots,m, $$

*and*

5.2 $$\begin{aligned} \begin{aligned} &\frac{(i+t_{3})^{s_{3}}H_{m,t_{3},s_{3}}}{(i+t_{2})^{s_{2}}H_{m,t_{2},s_{2}}} ={ \sum _{j=1}^{m}} \frac {(j+t_{3})^{s_{3}}H_{m,t_{3},s_{3}}}{(j+t_{1})^{s_{1}}H_{m,t_{1},s_{1}}}a_{ij},\quad i=1,\ldots,m, \\ &\frac{1}{(j+t_{3})^{s_{3}}H_{m,t_{3},s_{3}}} ={ \sum _{i=1}^{m}} \frac{1}{(i+t_{3})^{s_{3}}H_{m,t_{3},s_{3}}}a_{ij},\quad j=1,\ldots,m, \end{aligned} \end{aligned}$$

*hold for some row stochastic matrix* $A=(a_{ij})\in\mathcal{M}_{m}(\mathbb{R})$. *Then*, *for every* 4-*convex function* $\phi:[\alpha,\beta ]\rightarrow\mathbb{R}$, *we have*

5.3 $$\begin{aligned} 0 & \leq\widetilde{I}_{\phi} ( i,m,t_{1},t_{3},s_{1},s_{3} ) -\widetilde{I}_{\phi} ( i,m,t_{2},t_{3},s_{2},s_{3} ) - \int _{\alpha}^{\beta}\mathcal{G}(y) \bigl( \phi^{\prime\prime}(\alpha)+\phi^{\prime \prime\prime}(\alpha) ( y-\alpha ) \bigr)\,dy \\ & \leq \bigl\Vert \phi^{(4)} \bigr\Vert _{p} \biggl( \int_{\alpha}^{\beta } \biggl( \int_{\alpha}^{\beta}\mathcal{G}(y)G_{2, 1} ( y,u ) \,dy \biggr) ^{q}\,du \biggr) ^{\frac{1}{q}}, \end{aligned}$$

*where*

5.4 $$\begin{aligned} \mathcal{G}(y) ={}&{ \sum _{i=1}^{m}} \frac{1}{ ( i+t_{3} ) ^{s_{3}}H_{m,t_{3},s_{3}}}G \biggl( \frac {(i+t_{3})^{s_{3}}H_{m,t_{3},s_{3}}}{(i+t_{1})^{s_{1}}H_{m,t_{1},s_{1}}},y \biggr) \\ &{} -{ \sum_{i=1}^{m}} \frac{1}{ ( i+t_{3} ) ^{s_{3}}H_{m,t_{3},s_{3}}}G \biggl( \frac {(i+t_{3})^{s_{3}}H_{m,t_{3},s_{3}}}{(i+t_{2})^{s_{2}}H_{m,t_{2},s_{2}}},y \biggr). \end{aligned}$$

### Proof

If we substitute $x_{i}$ by $\frac{(i+t_{3})^{s_{3}}H_{m,t_{3},s_{3}}}{(i+t_{1})^{s_{1}}H_{m,t_{1},s_{1}}}$, $y_{i}$ by $\frac {(i+t_{3})^{s_{3}}H_{n,t_{3},s_{3}}}{(i+t_{2})^{s_{2}}H_{m,t_{2},s_{2}}}$, and $a_{i}$ by $\frac{1}{(i+t_{3})^{s_{3}}H_{m,t_{3},s_{3}}}$ in (3.9), we obtain the required result. □

### Corollary 9

*Let* $p,q$ *be a pair of conjugate exponents*, *i*.*e*., $1< p,q<\infty$, $1/p+1/q=1$. *Let* *G*, $G_{2, 1}$ *be defined by* (2.1), (3.8), *respectively*. *Let* $m\in\mathbb{N}$, $t_{1},t_{2} \geq0$ *and* $s_{1},s_{2},r_{1},\ldots,r_{m}>0$ *be such that*

5.5 $$ \frac{1}{r_{i}(i+t_{1})^{s_{1}}H_{m,t_{1},s_{1}}}, \frac{1}{r_{i}(i+t_{2})^{s_{2}}H_{m,t_{2},s_{2}}}\in{}[\alpha,\beta],\quad i=1,\ldots,m $$

*and*

5.6 $$\begin{aligned} \begin{aligned} &\frac{1}{r_{i}(i+t_{2})^{s_{2}}H_{m,t_{2},s_{2}}} ={ \sum _{j=1}^{m}} \frac{1}{r_{j}(j+t_{1})^{s_{1}}H_{m,t_{1},s_{1}}}a_{ij},\quad i=1,\ldots,m, \\ &r_{j} ={ \sum _{i=1}^{m}} r_{i}a_{ij},\quad j=1,\ldots,m, \end{aligned} \end{aligned}$$

*hold for some row stochastic matrix* $A=(a_{ij})\in\mathcal{M}_{m}(\mathbb{R})$. *Then*, *for every* 4-*convex function* $\phi:[\alpha,\beta ]\rightarrow\mathbb{R}$, *we have*

5.7 $$\begin{aligned} 0 & \leq\hat{I}_{\phi} ( m,t_{1},s_{1},\mathbf{r} ) -\hat {I}_{\phi} ( m,t_{2},s_{2},\mathbf{r} ) - \int_{\alpha}^{\beta }\mathcal{G}(y) \bigl( \phi^{\prime\prime}(\alpha)+\phi^{\prime\prime\prime}(\alpha) ( y-\alpha ) \bigr)\,dy \\ & \leq \bigl\Vert \phi^{(4)} \bigr\Vert _{p} \biggl( \int_{\alpha}^{\beta } \biggl( \int_{\alpha}^{\beta}\mathcal{G}(y)G_{2, 1} ( y,u ) \,dy \biggr) ^{q}\,du \biggr) ^{\frac{1}{q}}, \end{aligned}$$

*where*

5.8 $$ \mathcal{G}(y)={ \sum _{i=1}^{m}} r_{i}G \biggl( \frac{1}{r_{i}(i+t_{1})^{s_{1}}H_{m,t_{1},s_{1}}},y \biggr) -{ \sum _{i=1}^{m}} r_{i}G \biggl( \frac{1}{r_{i}(i+t_{2})^{s_{2}}H_{m,t_{2},s_{2}}},y \biggr). $$

### Proof

If we substitute $x_{i}$ by $\frac {1}{r_{i}(i+t_{1})^{s_{1}}H_{m,t_{1},s_{1}}}$, $y_{i}$ by $\frac{1}{r_{i}(i+t_{2})^{s_{2}}H_{m,t_{2},s_{2}}}$, and $a_{i}$ by $r_{i}>0$ in (3.9), we obtain the required result. □

### Corollary 10

*Let* $p,q$ *be a pair of conjugate exponents*, *i*.*e*., $1< p,q<\infty$, $1/p+1/q=1$. *Let* *G*, $G_{2, 1}$ *be defined by* (2.1), (3.8), *respectively*. *Let* $m\in\mathbb{N}$, $t_{1},t_{2} \geq0$, *and* $s_{1},s_{2}>0$ *be such that*

5.9 $$ \frac{1}{(i+t_{1})^{s_{1}}H_{m,t_{1},s_{1}}}, \frac{1}{(i+t_{2})^{s_{2}}H_{m,t_{2},s_{2}}}\in{}[\alpha,\beta],\quad i=1,\ldots,m $$

*and*

5.10 $$ \frac{1}{(i+t_{2})^{s_{2}}H_{m,t_{2},s_{2}}}={ \sum _{j=1}^{m}} \frac{1}{(j+t_{1})^{s_{1}}H_{m,t_{1},s_{1}}}a_{ij},\quad i=1,\ldots,m, $$

*hold for some row stochastic matrix* $A=(a_{ij})\in\mathcal{M}_{m}(\mathbb{R})$. *Then*, *for every* 4-*convex function* $\phi:[\alpha,\beta ]\rightarrow\mathbb{R}$, *we have*

$$\begin{aligned} 0 & \leq\hat{I}_{\phi} ( m,t_{1},s_{1},\mathbf{1} ) -\hat {I}_{\phi} ( m,t_{2},s_{2},\mathbf{1} ) - \int_{\alpha}^{\beta }\mathcal{G}(y) \bigl( \phi^{\prime\prime}(\alpha)+\phi^{\prime\prime\prime}(\beta) ( y-\alpha ) \bigr)\,dy \\ & \leq \bigl\Vert \phi^{(4)} \bigr\Vert _{p} \biggl( \int_{\alpha}^{\beta } \biggl( \int_{\alpha}^{\beta}\mathcal{G}(y)G_{2, 1} ( y,u ) \,dy \biggr) ^{q}\,du \biggr) ^{\frac{1}{q}}, \end{aligned}$$

*where*

5.11 $$ \mathcal{G}(y)={ \sum _{i=1}^{m}} G \biggl( \frac{1}{(i+t_{1})^{s_{1}}H_{m,t_{1},s_{1}}},y \biggr) -{ \sum_{i=1}^{m}} G \biggl( \frac{1}{(i+t_{2})^{s_{2}}H_{m,t_{2},s_{2}}},y \biggr). $$

### Proof

Substituting $r_{i}=1,i=1,\ldots,m$, in (5.7), we get the required result. □

Next we introduce definitions of Shannon’s entropy for the Zipf–Mandelbrot law.

### Definition 2

(Shannon’s entropy for Z–M law)

Let $m\in\mathbb{N}$.

- For $t \geq0$ and $s,r_{1},\ldots,r_{m}>0$, we define
  $$\hat{H} ( m,t,s,\mathbf{r} ) =-\sum_{i=1}^{m}r_{i} \ln \biggl( \frac{1}{r_{{i}}(i+t)^{s}H_{m,t,s}} \biggr). $$
- For $t, \tilde{t} \geq0$ and $s,\tilde{s}>0$, we define
  $$\widetilde{H} ( m,t,\tilde{t},s,\tilde{s} ) =-\sum_{i=1}^{m}\frac{1}{(i+\tilde{t})^{\tilde{s}}H_{m,\tilde{t},\tilde{s}}}\ln \biggl( \frac{(i+\tilde{t})^{\tilde{s}}H_{m,\tilde{t},\tilde {s}}}{(i+t)^{s}H_{m,t,s}} \biggr). $$

### Corollary 11

*Let* $p,q$ *be a pair of conjugate exponents*, *i*.*e*., $1< p,q<\infty$, $1/p+1/q=1$. *Let* $G_{2, 1}$ *be defined by* (3.8) *and* $m\in\mathbb{N}$.

(i) *If* $t_{1},t_{2},t_{3} \geq0$ *and* $s_{1},s_{2},s_{3}>0$ *are such that* (5.1) *and* (5.2) *hold for some row stochastic matrix* $A=(a_{ij})\in\mathcal{M}_{m}(\mathbb{R})$, *then*
  $$\begin{aligned} 0&\leq\widetilde{H} ( m,t_{1},t_{3},s_{1},s_{3} ) -\widetilde{H} ( m,t_{2},t_{3},s_{2},s_{3} ) - \int_{\alpha }^{\beta }\mathcal{G}(y)\frac{3\alpha-2y}{\alpha^{3}}\,dy \\ & \leq6 \biggl( \frac{\beta^{1-4p}-\alpha^{1-4p}}{1-4p} \biggr) ^{\frac{1}{p}} \biggl( \int_{\alpha}^{\beta} \biggl( \int_{\alpha}^{\beta}\mathcal{G}(y)G_{2, 1} ( y,u ) \,dy \biggr) ^{q}\,du \biggr) ^{\frac{1}{q}}, \end{aligned}$$
  *where* $\mathcal{G}(y)$ *is defined by* (5.4).
(ii) *If* $t_{1},t_{2} \geq0$ *and* $s_{1},s_{2},r_{1},\ldots,r_{m}>0$ *are such that* (5.5) *and* (5.6) *hold for some row stochastic matrix* $A=(a_{ij})\in\mathcal{M}_{m}(\mathbb{R})$, *then*
  5.12 $$\begin{aligned} 0&\leq\hat{H} ( m,t_{1},s_{1},\mathbf{r} ) -\hat{H} ( m,t_{2},s_{2},\mathbf{r} ) - \int_{\alpha}^{\beta}\mathcal{G}(y) \frac{3\alpha-2y}{\alpha^{3}}\,dy \\ & \leq6 \biggl( \frac{\beta^{1-4p}-\alpha^{1-4p}}{1-4p} \biggr) ^{\frac{1}{p}} \biggl( \int_{\alpha}^{\beta} \biggl( \int_{\alpha}^{\beta}\mathcal{G}(y)G_{2, 1} ( y,u ) \,dy \biggr) ^{q}\,du \biggr) ^{\frac{1}{q}}, \end{aligned}$$
  *where* $\mathcal{G}(y)$ *is defined by* (5.8).
(iii) *If* $t_{1},t_{2} \geq0$ *and* $s_{1},s_{2}>0$ *are such that* (5.9) *and* (5.10) *hold for some row stochastic matrix* $A=(a_{ij})\in\mathcal{M}_{m}(\mathbb{R})$, *then*
  $$\begin{aligned} 0&\leq\hat{H} ( m,t_{1},s_{1},\mathbf{1} ) -\hat{H} ( m,t_{2},s_{2},\mathbf{1} ) - \int_{\alpha}^{\beta}\mathcal{G}(y) \frac{3\alpha-2y}{\alpha^{3}}\,dy \\ & \leq6 \biggl( \frac{\beta^{1-4p}-\alpha^{1-4p}}{1-4p} \biggr) ^{\frac{1}{p}} \biggl( \int_{\alpha}^{\beta} \biggl( \int_{\alpha}^{\beta}\mathcal{G}(y)G_{2, 1} ( y,u ) \,dy \biggr) ^{q}\,du \biggr) ^{\frac{1}{q}}, \end{aligned}$$
  *where* $\mathcal{G}(y)$ *is defined by* (5.11).

### Proof

(i) Substituting $x_{i}$ by $\frac{(i+t_{3})^{s_{3}}H_{m,t_{3},s_{3}}}{(i+t_{1})^{s_{1}}H_{m,t_{1},s_{1}}}$, $y_{i}$ by $\frac {(i+t_{3})^{s_{3}}H_{n,t_{3},s_{3}}}{(i+t_{2})^{s_{2}}H_{m,t_{2},s_{2}}}$, and $a_{i}$ by $\frac{1}{(i+t_{3})^{s_{3}}H_{m,t_{3},s_{3}}}$ and taking $\phi(x)=-\ln x$ in (3.9), we get the required result.

(ii) Substituting $x_{i}$ by $\frac{1}{r_{i}(i+t_{1})^{s_{1}}H_{m,t_{1},s_{1}}}$, $y_{i}$ by $\frac {1}{r_{i}(i+t_{2})^{s_{2}}H_{m,t_{2},s_{2}}}$, $a_{i}$ by $r_{i}>0$ in (3.9) and taking $\phi(x)=-\ln x$, we get the required result.

(iii) Substituting $r_{i}=1$ in (5.12), we get the required result. □

At the end, we introduce the Kullback–Leibler divergence for the Zipf–Mandelbrot law. For more information about the Kullback–Leibler divergence, see [22, 23].

### Definition 3

(The Kullback–Leibler divergence for Z–M)

Let $m\in\mathbb{N}$.

- For $t \geq0$ and $s,r_{1},\ldots,r_{m}>0$, we define
  $$\hat{KL} ( m,t,s,\mathbf{r} ) =\sum_{i=1}^{m} \frac{1}{(i+t)^{s}H_{m,t,s}}\ln \biggl( \frac{1}{r_{{i}}(i+t)^{s}H_{m,t,s}} \biggr). $$
  Specially, when $r_{i}=1$, $i=1,\ldots,m$, we have
  $$\hat{KL} ( m,t,s,\mathbf{1} ) =\sum_{i=1}^{m} \frac{1}{(i+t)^{s}H_{m,t,s}}\ln \biggl( \frac{1}{(i+t)^{s}H_{m,t,s}} \biggr). $$
- For $t, \tilde{t} \geq0$ and $s,\tilde{s}>0$, we define
  $$\widetilde{KL} ( m,t,\tilde{t},s,\tilde{s} ) =\sum_{i=1}^{m}\frac{1}{(i+t)^{s}H_{m,t,s}}\ln \biggl( \frac{(i+\tilde{t})^{\tilde{s}}H_{m,\tilde{t},\tilde{s}}}{(i+t)^{s}H_{m,t,s}} \biggr). $$

### Corollary 12

*Let* $p,q$ *be a pair of conjugate exponents*, *i*.*e*., $1< p,q<\infty$, $1/p+1/q=1$. *Let* $G_{2, 1}$ *be defined by* (3.8) *and* $m\in\mathbb{N}$.

(i) *If* $t_{1},t_{2},t_{3} \geq0$ *and* $s_{1},s_{2},s_{3}>0$ *are such that* (5.1) *and* (5.2) *hold for some row stochastic matrix* $A=(a_{ij})\in\mathcal{M}_{m}(\mathbb{R})$, *then*
  $$\begin{aligned} 0&\leq\widetilde{KL} ( m,t_{1},t_{3},s_{1},s_{3} ) -\widetilde{KL} ( m,t_{2},t_{3},s_{2},s_{3} ) - \int_{\alpha}^{\beta}\mathcal{G}(y) \frac{2\alpha-y}{\alpha^{2}}\,dy \\ & \leq2 \biggl( \frac{\beta^{1-3p}-\alpha^{1-3p}}{1-3p} \biggr) ^{\frac{1}{p}} \biggl( \int_{\alpha}^{\beta} \biggl( \int_{\alpha}^{\beta}\mathcal{G}(y)G_{2, 1} ( y,u ) \,dy \biggr) ^{q}\,du \biggr) ^{\frac{1}{q}}, \end{aligned}$$
  *where* $\mathcal{G}(y)$ *is defined by* (5.4).
(ii) *If* $t_{1},t_{2} \geq0$ *and* $s_{1},s_{2},r_{1},\ldots,r_{m}>0 $ *are such that* (5.5) *and* (5.6) *hold for some row stochastic matrix* $A=(a_{ij})\in\mathcal{M}_{m}(\mathbb{R})$, *then*
  5.13 $$\begin{aligned} 0&\leq\hat{KL} ( m,t_{1},s_{1},\mathbf{r} ) -\hat {KL} ( m,t_{2},s_{2},\mathbf{r} ) - \int_{\alpha}^{\beta }\mathcal {G}(y)\frac{2\alpha-y}{\alpha^{2}} \,dy \\ & \leq2 \biggl( \frac{\beta^{1-3p}-\alpha^{1-3p}}{1-3p} \biggr) ^{\frac{1}{p}} \biggl( \int_{\alpha}^{\beta} \biggl( \int_{\alpha}^{\beta}\mathcal{G}(y)G_{2, 1} ( y,u ) \,dy \biggr) ^{q}\,du \biggr) ^{\frac{1}{q}}, \end{aligned}$$
  *where* $\mathcal{G}(y)$ *is defined by* (5.8).
(iii) *If* $t_{1},t_{2} \geq0$ *and* $s_{1},s_{2}>0$ *are such that* (5.9) *and* (5.10) *hold for some row stochastic matrix* $A=(a_{ij})\in\mathcal{M}_{m}(\mathbb{R})$, *then*
  $$\begin{aligned} 0&\leq\hat{KL} ( m,t_{1},s_{1},\mathbf{1} ) -\hat {KL} ( m,t_{2},s_{2},\mathbf{1} ) - \int_{\alpha}^{\beta }\mathcal {G}(y)\frac{2\alpha-y}{\alpha^{2}}\,dy \\ & \leq2 \biggl( \frac{\beta^{1-3p}-\alpha^{1-3p}}{1-3p} \biggr) ^{\frac{1}{p}} \biggl( \int_{\alpha}^{\beta} \biggl( \int_{\alpha}^{\beta}\mathcal{G}(y)G_{2, 1} ( y,u ) \,dy \biggr) ^{q}\,du \biggr) ^{\frac{1}{q}}, \end{aligned}$$
  *where* $\mathcal{G}(y)$ *is defined by* (5.11).

### Proof

(i) Substituting $x_{i}$ by $\frac{(i+t_{3})^{s_{3}}H_{m,t_{3},s_{3}}}{(i+t_{1})^{s_{1}}H_{m,t_{1},s_{1}}}$, $y_{i}$ by $\frac {(i+t_{3})^{s_{3}}H_{n,t_{3},s_{3}}}{(i+t_{2})^{s_{2}}H_{m,t_{2},s_{2}}}$, $a_{i}$ by $\frac {1}{(i+t_{3})^{s_{3}}H_{m,t_{3},s_{3}}}$ in (3.9) and taking $\phi(x)=x\ln x$, we get the required result.

(ii) Substituting $x_{i}$ by $\frac{1}{r_{i}(i+t_{1})^{s_{1}}H_{m,t_{1},s_{1}}}$, $y_{i}$ by $\frac {1}{r_{i}(i+t_{2})^{s_{2}}H_{m,t_{2},s_{2}}}$, and $a_{i}$ by $r_{i}$ for each $i=1,\ldots,m$ in (3.9) and taking $\phi(x)=x\ln x$, we get the required result.

(iii) Substituting $r_{i}=1,i=1,\ldots,m$, in (5.13), we get the required result. □

## Conclusions

In this paper we have given generalized results for Sherman’s inequality by considering the class of convex functions of higher order. We obtained an extended weighted majorization inequality as well as Jensen’s inequality as special cases directly connected to information theory. We used the obtained results to derive new estimates for Shannon’s and Rényi’s entropy, information energy, and some well-known measures between probability distributions. Using the Zipf–Mandelbrot law, we introduced new functionals to derive some related results.
